# Supplementary material for: Second-Generation Antipsychotics’ Effectiveness and Tolerability: A Review of Real-World Studies in Patients with Schizophrenia and Related Disorders
Source: J Clin Med. 2022 Aug 3;11(15):4530. doi: 10.3390/jcm11154530 (PMC9369504; doi:10.3390/jcm11154530)
Supplement: Supplementary file 1 [file jcm-11-04530-s001.zip › jcm-1793077-supplementary-Table S1.pdf]

Supplemetary Table S1. Risk of bias assessments in non-randomized clinical studies

Authors (year of publication)	Type of study	Pre-intervention domains		At-intervention domain	Post-intervention domains				Overall Risk of Bias
		Confounding bias	*Selection bias*	*Information bias*	*Confounding bias*	*Selection bias*	*Information bias*	*Reporting bias*	
Alphs et al. (2015)	Open-label study	High	High	High	Low	Low	Moderate	Low	**High**
Barbosa et al. (2021)	Cohort study	Moderate	Low	High	Low	Low	High	Moderate	**High**
Chan et al. (2015)	Cohort study	High	Moderate	High	Moderate	Low	High	Moderate	**High**
Cortesi et al. (2013)	Cohort study	Low	Low	Low	Low	Moderate	Moderate	Low	**Moderate**
Devrimci-Ozguven et al. (2019)	Image study	High	Low	Moderate	Moderate	Low	Low	Moderate	**High**
Fagiolini et al. (2019)	Observational study	Low	Moderate	Low	Low	Low	Moderate	Low	**Moderate**
Fernández-Miranda et al. (2017)	Prospective observational study	High	High	Low	Moderate	Moderate	Low	Low	**High**
Fernández-Miranda et al. (2020)	Observational mirror-image study	Moderate	Moderate	Low	Moderate	Low	Low	Low	**Moderate**
Fernández-Miranda et al. (2021)	Observational study	High	Low	Moderate	Moderate	Low	Moderate	Low	**High**
Hargarter et al. (2015)	Open-label study	Moderate	Low	Low	Low	Moderate	Low	Low	**Moderate**
Hatta et al. (2022)	Cohort study	High	Low	High	Low	Moderate	High	Moderate	**High**
Iqbal et al. (2020)		Moderate	Low	High	Low	Low	High	Moderate	**High**
Iwata et al. (2020)	Cohort study	High	Low	Moderate	Low	Low	High	Moderate	**High**
Kilzieh et al. (2008)	Retrospective study	Moderate	Low	Low	Low	Moderate	Low	Low	**Moderate**
Lauriello et al. (2021)	Cohort study	Moderate	Low	Low	Low	Low	Moderate	Low	**Moderate**
Magliocco et al. (2020)	Longitudinal prospective study	Moderate	Low	Moderate	Low	Moderate	Moderate	Moderate	**Moderate**
Mahabaleshwarkar et al. (2021)	Mirror image study	Moderate	Low	High	Low	Low	High	Moderate	**High**
Misawa et al. (2017)	Mirror image study	Moderate	Moderate	Moderate	Moderate	Low	Moderate	Moderate	**Moderate**
Novick et al. (2016)	Observational study	Low	Low	Low	Low	Low	Low	Low	**Low**

Patel et al. (2019)	Retrospective claims-based study	Moderate	Low	Moderate	Low	Low	Moderate	Low	**Moderate**
Pilon et al (2017)	Cohort study	High	Low	Low	Moderate	Moderate	Moderate	Moderate	**High**
Rajagopalan et al. (2017)	Database	Low	Low	Moderate	Low	Low	Moderate	Low	**Moderate**
Ratner et al. (2007)	Observational trial	Moderate	Moderate	Low	Low	Low	Low	Low	**Moderate**
Ritsner et al (2007)	Observational trial	Moderate	Moderate	Low	Low	Low	Low	Low	**Moderate**
Rosso et al. (2016)	Observational study	Low	Moderate	Low	Moderate	Moderate	Moderate	Low	**Moderate**
Schöttle et al. (2018)	Non interventional study	Low	Moderate	Moderate	Low	Moderate	High	Moderate	**High**
Schreiner et al. (2014)	Interventional unblinded study	Moderate	Low	Moderate	Low	Moderate	Moderate	Low	**Moderate**
Stam et al. (2020)	Database	Moderate	Low	Low	Moderate	Low	Moderate	Moderate	**Moderate**
Takàcs et al. (2019)	Longitudinal study	Moderate	Moderate	Moderate	Low	Low	Moderate	Moderate	**Moderate**
Taylor et al. (2005)	Prospective comparative outcome study	Moderate	Moderate	Moderate	Low	Moderate	Moderate	Moderate	**Moderate**
Tiihonen et al. (2017)	Cohort study	Moderate	Moderate	Low	Moderate	Low	Moderate	Moderate	**Moderate**
Vanasse et al. (2016)	Cohort study	Moderate	Low	Moderate	Low	Low	Moderate	Low	**Moderate**
Yan et al. (2020)	Cohort study	High	Moderate	High	Low	Low	Moderate	Low	**High**
Zhang et al. (2019)	Real-world clinical study	Low	Moderate	Low	Moderate	Low	Moderate	Low	**Moderate**
