# Supplementary figures and images for: Second-Generation Antipsychotics’ Effectiveness and Tolerability: A Review of Real-World Studies in Patients with Schizophrenia and Related Disorders
Source: J Clin Med. 2022 Aug 3;11(15):4530. doi: 10.3390/jcm11154530 (PMC9369504; doi:10.3390/jcm11154530)

**Supplementary Figure S1.** Flow diagram showing study selection process of included articles.

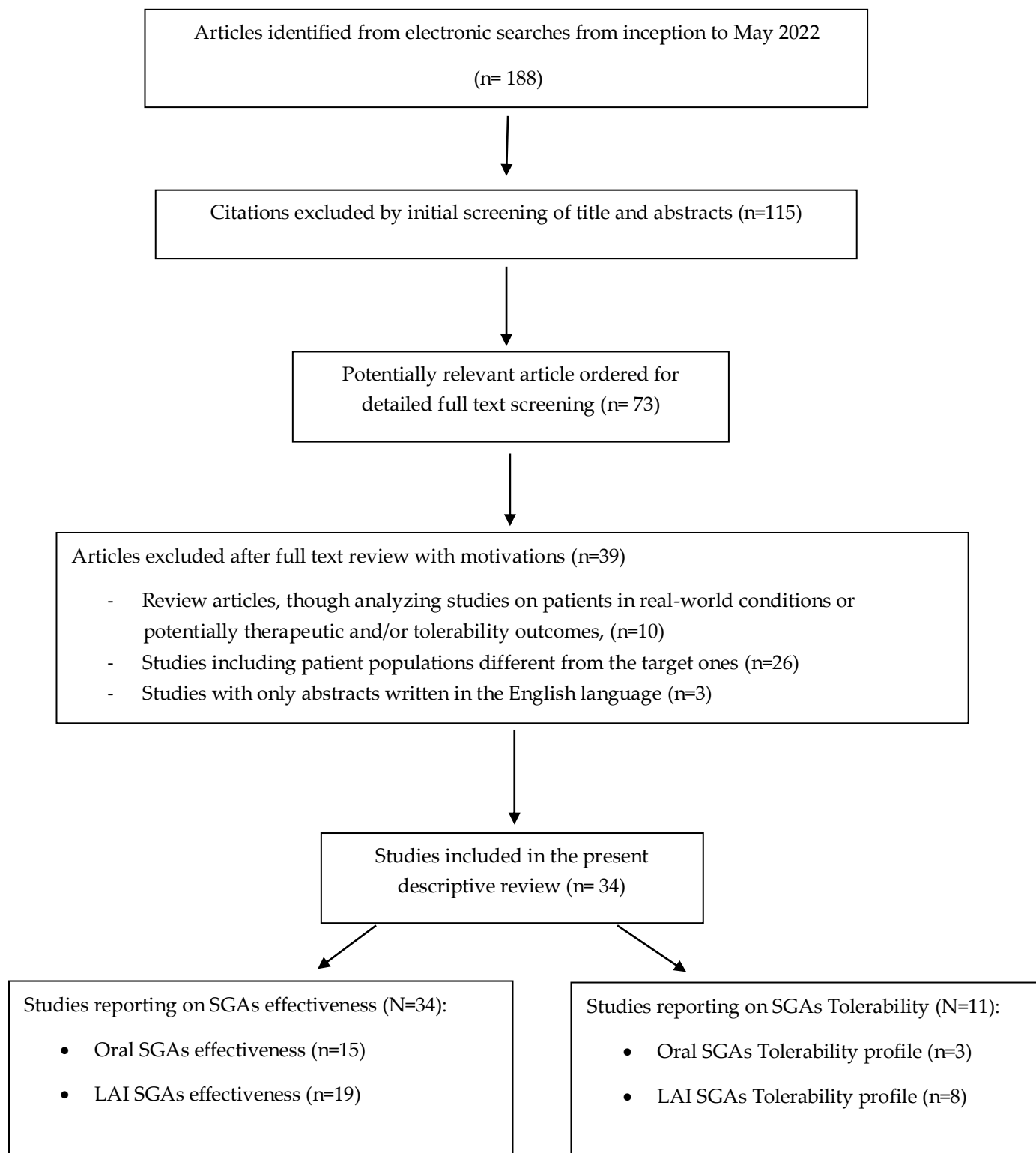

Supplement: Supplementary file 1 [file jcm-11-04530-s001.zip › jcm-1793077-supplementary-Figure S1.pdf]
